# Multiple aspects of male germ cell development and interactions with Sertoli cells require inositol hexakisphosphate kinase-1

**DOI:** 10.1038/s41598-018-25468-8

**Published:** 2018-05-04

**Authors:** Chenglai Fu, Tomas Rojas, Alfred C. Chin, Weiwei Cheng, Isaac A. Bernstein, Lauren K. Albacarys, William W. Wright, Solomon H. Snyder

**Affiliations:** 1 Department of Physiology and Pathophysiology, Tianjin Medical University, Tianjin, 300070 China; 2 The Solomon H. Snyder Department of Neuroscience, Johns Hopkins University School of Medicine, Baltimore, MD 21205 USA; 3 Division of Neuropathology, Department of Pathology, Johns Hopkins University School of Medicine, Baltimore, MD 21205 USA; 4 Department of Biochemistry and Molecular Biology, Johns Hopkins Bloomberg School of Public Health, Baltimore, MD 21205 USA; 5 Department of Pharmacology and Molecular Sciences, Johns Hopkins University School of Medicine, Baltimore, MD 21205 USA; 6 Department of Psychiatry and Behavioral Sciences, Johns Hopkins University School of Medicine, Baltimore, MD 21205 USA

## Abstract

Inositol hexakisphosphate kinase-1 (IP6K1) is required for male fertility, but the underlying mechanisms have been elusive. Here, we report that IP6K1 is required for multiple aspects of male germ cell development. This development requires selective interactions between germ cells and Sertoli cells, namely apical ectoplasmic specialization. Spermiation (sperm release) requires tubulobulbar complexes. We found that the apical ectoplasmic specialization and tubulobulbar complexes were poorly formed or disrupted in *IP6K1* KOs. Deletion of IP6K1 elicited several aberrations, including: 1, sloughing off of round germ cells; 2, disorientation and malformation of elongating/elongated spermatids; 3, degeneration of acrosomes; 4, defects in germ-Sertoli cell interactions and 5, failure of spermiation. Eventually the sperm cells were not released but phagocytosed by Sertoli cells leading to an absence of sperm in the epididymis.

## Introduction

Inositol hexakisphosphate kinase (IP6K) comprises a family of three kinases, namely IP6K1, IP6K2 and IP6K3^1–3^. Functions of IP6Ks have been inferred from studies of mice with targeted deletion of IP6K1, IP6K2 and IP6K3. IP6K1 is involved in diverse functions. It determines insulin release^4^, regulates cell migration^5,6^, histone demethylation^7^ and cell metabolisms^8–10^. Important for this study, it was reported that *IP6K1* KO male mice are infertile with no sperm in the epididymides^1,11^. Recently, Bhandari and associates reported that IP6K1 is essential for the formation of the chromatoid body^11^. They found that the chromatoid body is absent in *IP6K1* KO round spermatids, and the mutant spermatids failed to complete differentiation^11^. However, despite these advances, the influence of IP6K1 upon male germ cell development has not been fully delineated.

Spermatogenesis, the generation of motile sperm cells from spermatogonial stem cells, is largely regulated by follicle stimulating hormone (FSH) and luteinizing hormone (LH) released from the pituitary gland as well as testosterone produced by Leydig cells. The microenvironment of the seminiferous epithelium also is critical for sperm development^12^. In seminiferous tubules, germ cells are surrounded by Sertoli cells, which orchestrate the organization of testicular structures. Sertoli cells are “nurse” cells that influence the survival, replication, differentiation, and movement of germ cells via direct contact and by controlling the environment milieu within the seminiferous tubules^13^. Sertoli cells provide physical and nutritional support for germ cells^14,15^ as well as secreting factors to control maintenance and self-renewal of spermatogonial stem cells^16^. Sertoli cells protect germ cells by forming the blood-testis barrier and expressing immunoregulatory factors, thereby creating a local tolerogenic environment optimal for survival of nonsequesetred auto-antigenic germ cells^17^. Sertoli cells also function as phagocytes to eliminate degenerated cells^18,19^.

In mice, spermatogenesis has been classified into 12 stages according to the arrangement of different germ cells that are developmentally synchronized in the seminiferous epithelium. In addition, the differentiation of round spermatids into mature, motile spermatozoa is divided into 16 steps. At step 16 mature, motile sperm are released (spermiation) corresponding to stage VII to VIII in spermatogenesis^20^.

Developing round and elongating/elongated spermatids must maintain stable attachments with Sertoli cells to prevent sloughing of immature germ cells from the seminiferous epithelium, which may result in infertility^21^. The apical ectoplasmic specialization (ES) is a unique adhesion junction formed between elongating/elongated spermatids and Sertoli cells, and is crucial for attachment of spermatids as well as their movement and orientation during spermatogenesis^22^. Disruption of germ cell-Sertoli cell interactions^23^ or spermatid polarization^24^ leads to spermatogenic defects and infertility. The molecular composition of apical ES includes integral membrane proteins, adaptor proteins, signaling proteins and cytoskeletal proteins. However, little is known about the regulation of apical ES dynamics^22,25^.

The apical tubulobulbar complex (TBC) is another linkage between maturing spermatids and Sertoli cells, formed by cytoplasmic processes extending from spermatids into the neighboring Sertoli cells^26^. In mice apical TBCs are transiently formed at spermatogenic stage VII prior to sperm release and function to remove excess cytoplasm from spermatids^27^ and to remove adhesion junctions between spermatids and Sertoli cells^26,28^. The apical TBC is comprised of a long proximal tubule followed by a bulb, then a short distal tubule and a coated pit^26^. Actin filaments are highly enriched in apical TBCs^29^. Defects of apical TBCs caused by chemical treatment or genetic mutation result in spermiation failure^30–32^.

In the present study we explored the roles of IP6K1 in male germ cell development. The elongating/elongated spermatids of *IP6K1* KO were disoriented and malformed. The acrosomes of *IP6K1* KO spermatids were also degenerated during differentiation. Defective apical ES and malformed apical TBCs were apparent in the *IP6K1* mutants. Eventually, the defective sperm cells failed to release and were engulfed by Sertoli cells at stage VII and stage VIII in the seminiferous epithelium of KO mice, which can account for the sperm deficits.

## Results

### Deletion of IP6K1 leads to male infertility caused by scarce and malformed sperms

**Figure 1:**
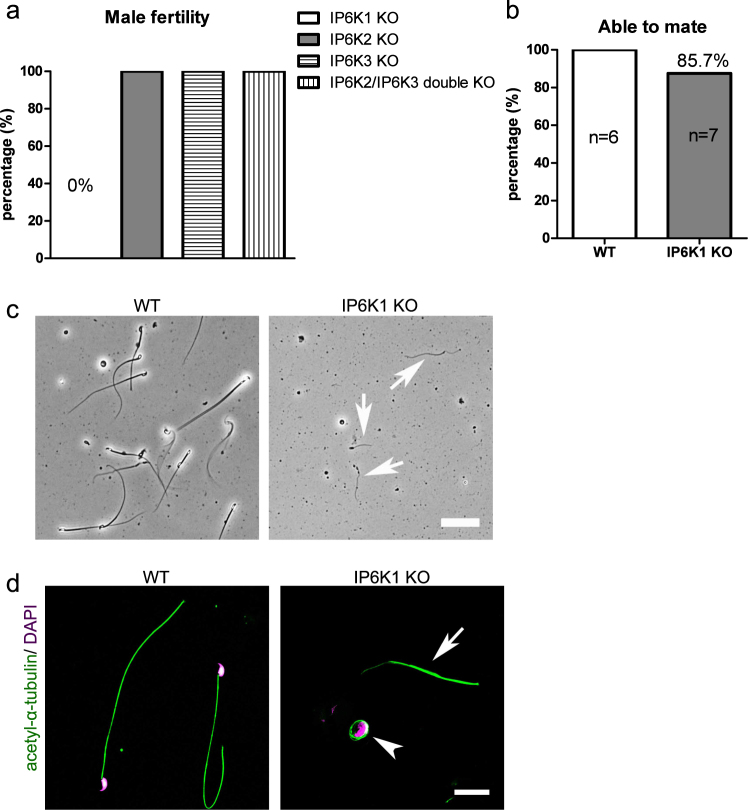
Infertility in IP6K1 deleted males was caused by scarce and malformed sperm. (**a**) None of *IP6K1* KO male mice (n = 7) impregnate wild type females. By contrast, *IP6K2* KO males (n = 6), *IP6K3* KO males (n = 6) and *IP6K2/IP6K3* double KO males (n = 6) are able to produce progeny. (**b**) *IP6K1* KO male mice (n = 7) were set to mate with wild type female mice, mating plugs were found in six of 7 wild type female mice. Six pairs of WT male and female mice were set as controls. (**c,d**) Sperm cells were isolated from epididymides. (**c**) Bright-field microscopy shows severed sperm tails (arrows) but no motile sperm cells in *IP6K1* KOs. Scale bar 50 μm. (**d**) Immunostaining of acetyl-α-tubulin for sperm tails. Nuclei were stained by DAPI. Severed sperm tails (arrow) and sperm heads coiled by sperm tails (arrowhead) were seen in the *IP6K1* mutants. Scale bar 20 μm.

Deletion of *IP6K1* in mice was shown to elicit male infertility^1,11^. In agreement with this finding, we observed a complete absence of offspring from IP6K1 deleted males, whereas fertility appeared normal in *IP6K1* KO females (Fig. S1a). In contrast to the lack of fertility of *IP6K1* KO males, mice with deletion of IP6K2, IP6K3 or double knockout of *IP6K2* and *IP6K3* displayed normal fertility (Fig. 1a). We also examined the sexual behavior and ability of mice to mate, which appeared to be normal in the *IP6K1* KO males (Fig. 1b).

Reproductive behavior and spermatogenesis is regulated by levels of reproductive hormones. However, plasma levels of FSH, LH and testosterone were normal in *IP6K1* KO male mice (Fig. S1b–d). Thus, the fertility defect of *IP6K1* KO male mice does not arise from hormonal deregulation.

We collected motile sperm cells from epididymides and observed very little cellular content and no motile sperm cells in the *IP6K1* KO mice (Fig. 1c). We observed severed sperm tails in the *IP6K1* KO preparations with some residual DNA surrounded by sperm tails (Figs 1d and S1e).

### Deletion of IP6K1 causes sloughing of round germ cells

**Figure 2:**
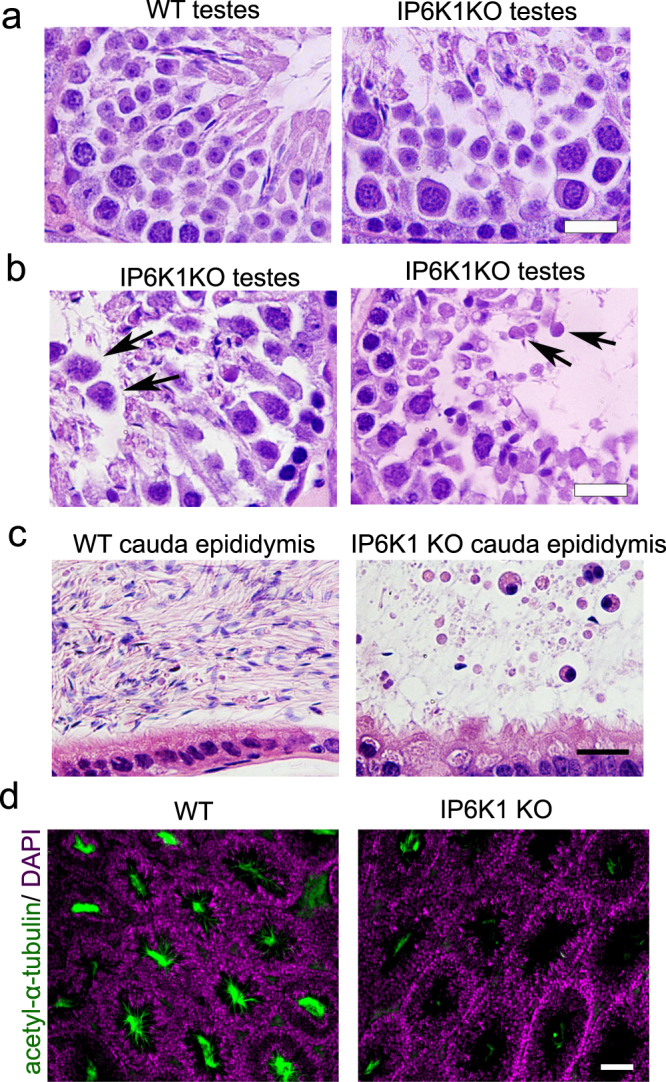
Deletion of IP6K1 elicited sloughing of round germ cells. (**a,b**) H&E staining of testes (**a**) Germ cells in *IP6K1* KO seminiferous tubules are more loosely connected to Sertoli cells. Scale bar 20 μm. (**b**) Round germ cells were sloughed off and appeared in the lumina of seminiferous tubules (arrows). Scale bar 20 μm. (**c**) H&E staining of cauda epididymides. Round germ cells were seen in *IP6K1* KO cauda epididymides. scale bar 20 μm. (**d**) Immunostaining of sperm tails in testes with anti acetyl-α-tubulin antibody; DAPI stains nucleus. Scale bar 100 μm.

We first looked at the gross morphology of the reproductive organs. Testes of the *IP6K1* KOs were reduced in size (Fig. S2a,b), and testicular weight was diminished by about 20 percent (Fig. S2b). We then looked at the microscopic morphology. In testes of *IP6K1* KOs, spermatids were more loosely attached to Sertoli cells than for wild-type animals (Fig. 2a), and round spermatids were dislodged (Fig. 2b). The sloughing of germ cells in *IP6K1* KO seminiferous tubules was confirmed by the presence of round cells in the cauda epididymides of *IP6K1* mutant mice (Fig. 2c). Thus, deletion of IP6K1 affected interactions between germ cells and Sertoli cells.

We examined sperm in testes by staining sperm tails for acetyl-α-tubulin, which revealed a virtually total loss of mature sperms in *IP6K1* KOs (Fig. 2d). In both caput and cauda epididymides of wild type animals, the lumens were typically filled with sperm cells. The lumens of *IP6K1* KO epididymides, however, were devoid of sperm, whereas levels in *IP6K2* and *IP6K3* KOs were comparable to wild-type animals (Fig. S2c,d).

### IP6K1 deletion causes malformation of elongating/elongated spermatids

**Figure 3:**
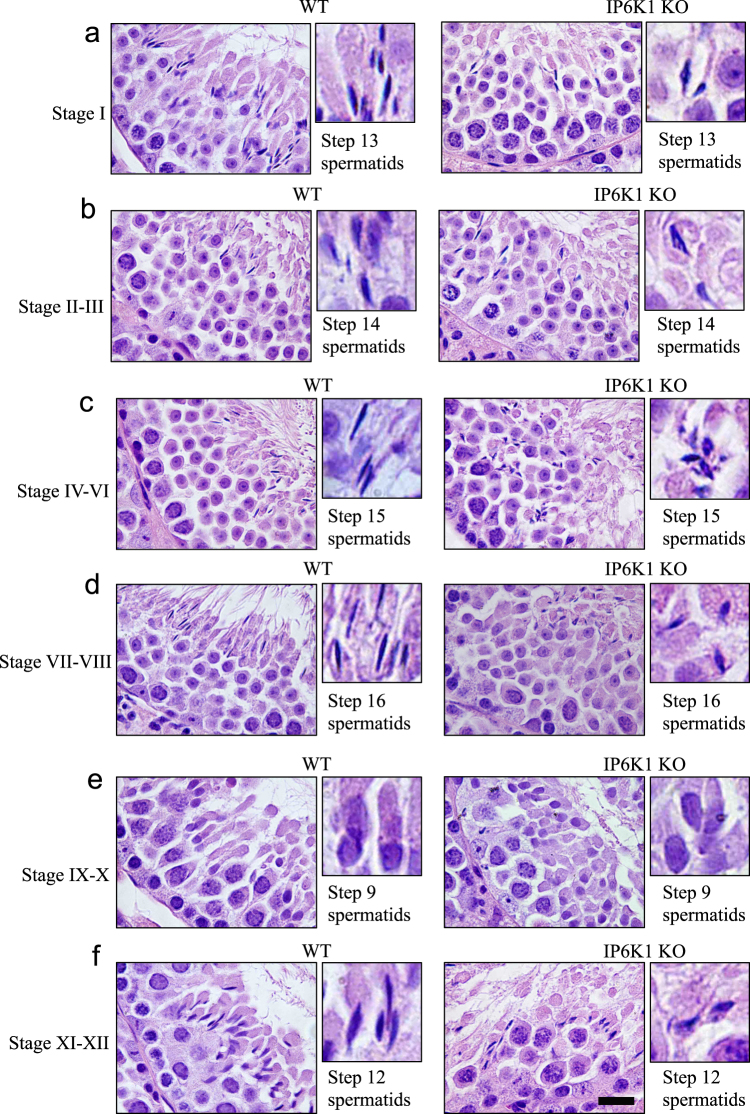
Deletion of IP6K1 elicited disorientation and malformation of elongating/elongated spermatids. (**a**–**f**) H&E staining of WT and *IP6K1* KO testes. Stages of seminiferous tubules were determined by spermatid developmental status. (**a**,**b**,**c**,**d**,**f**) Cell polarity and morphology of the step 12–16 elongating/elongated spermatids were disrupted in *IP6K1* KOs (insets). Scale bar 20 μm.

We examined the different stages of spermatogenesis in testes (Fig. 3). In *IP6K1* KOs, elongating/elongated spermatids displayed aberrant morphology. The steps 12–16 elongating/elongated spermatids of *IP6K1* mutants appeared disoriented and irregularly shaped (Fig. 3a–f). By contrast, no obvious defect was seen in steps 1–8 round spermatids in *IP6K1* KOs (Fig. S3).

Immunofluorescence staining of testes revealed abundant IP6K1 localized to the germ cells of wild-type mice, which was completely absent in *IP6K1* KOs (Fig. S4a). We noted major differences in the cellular localizations of IP6K1, IP6K2 and IP6K3 in testes (Fig. S4b). IP6K1 was ubiquitously expressed in germ cells; IP6K2 occurred in primary spermatocytes, while the expression of IP6K3 was low and only evident in step 12 spermatids (Fig. S4b). IP6K1 was expressed in elongating/elongated spermatids (Fig. S4c,d), implying that structural/functional defects in these cells in mutant mice stems from the loss of IP6K1 (Fig. 3). We explored a potential loss of Sertoli and Leydig cells in the *IP6K1* KOs. Staining of Sertoli cells for GATA4 and of Leydig cells with calretinin revealed relatively normal numbers of these cell types (Fig. S5a,b).

### IP6K1 deletion causes disorientation of spermatids and failure of spermiation

**Figure 4:**
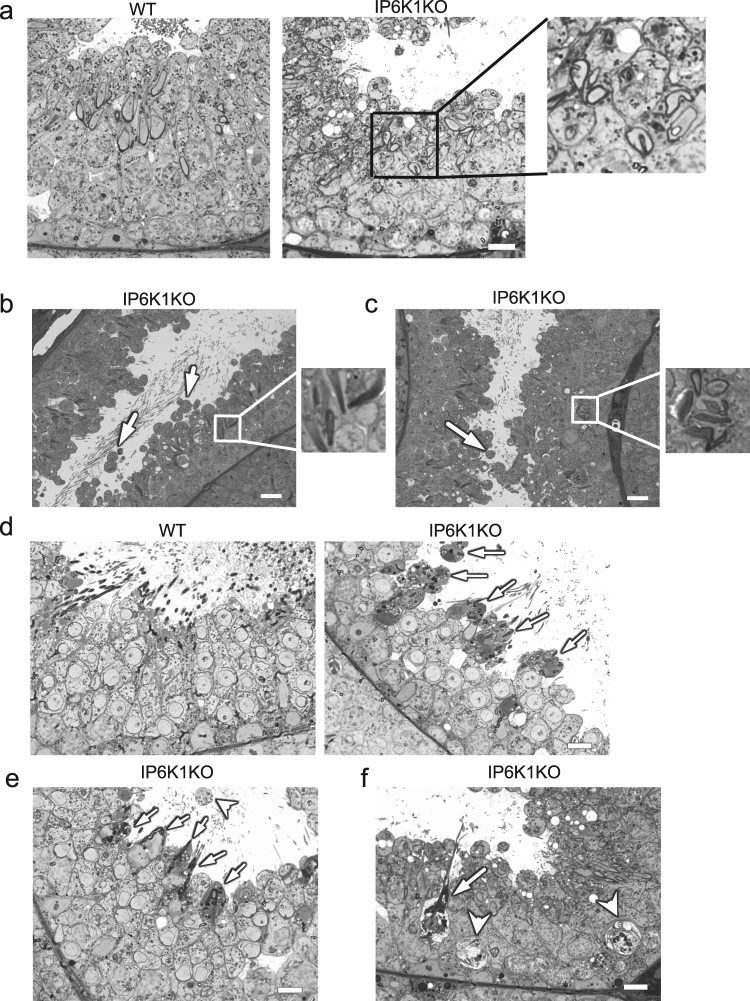
Spermiation failure and phagocytosis of sperm by Sertoli cells in *IP6K1* KOs. (**a**–**f**) Toluidine blue staining of testes. (**a**) In stage X seminiferous tubules of *IP6K1* KOs, the elongating spermatids were disoriented (inset). Scale bar 10 μm. (**b**) In stage I seminiferous tubules of *IP6K1* mutants, the elongated spermatids were disoriented (inset) and some round spermatids were sloughed off (arrows). Scale bar 20 μm. (**c**) In stage II-III seminiferous tubules of *IP6K1* KOs, the elongated spermatids were disoriented and malformed (inset), some round spermatids were dislodged (arrow). Scale bar 20 μm. (**d**) In stage VII seminiferous tubules. Sperm cells were not released but engulfed by Sertoli cells in *IP6K1* KOs (arrows). Scale bar 10 μm. (**e**) In stage VIII seminiferous tubules of *IP6K1* KOs, sperm cells were “eaten” by Sertoli cells (arrows). Sloughed off round spermatids were evident in the lumens (arrowhead). Scale bar 10 μm. (**f**) In stage IX seminiferous tubules of *IP6K1* mutants, “choking down” sperms killed Sertoli cells (arrow). Arrowheads point to phagosomes (digested spermatocytes) within Sertoli cells. Scale bar 10 μm.

We examined cellular morphology in the testes by Toluidine blue staining (Fig. 4). Disorientation, defined as a deviation of more than 90 degree from pointing to basement membrane perpendicular^33^, and abnormal morphology of elongating/elongated spermatids were pronounced in the mutants (Fig. 4a–c). Dislodged round spermatids were evident in the lumens of seminiferous tubules of *IP6K1* KOs (Fig. 4b,c and e). Most striking was the substantial engulfment of sperm by Sertoli cells (Fig. 4d–f). In wild type mice, the sperm were released at stage VII-VIII, and the lumens were full of sperm (Fig. 4d). However, sperm were not released in *IP6K1* KOs. Instead, they were “eaten” by Sertoli cells (Fig. 4d–f). The phagocytosis of sperm cells was prominent at stage VII (Fig. 4d) and stage VIII (Fig. 4e), when they should be released. The engulfed sperm cells were also observed at stage IX (Fig. 4f).

**Figure 5:**
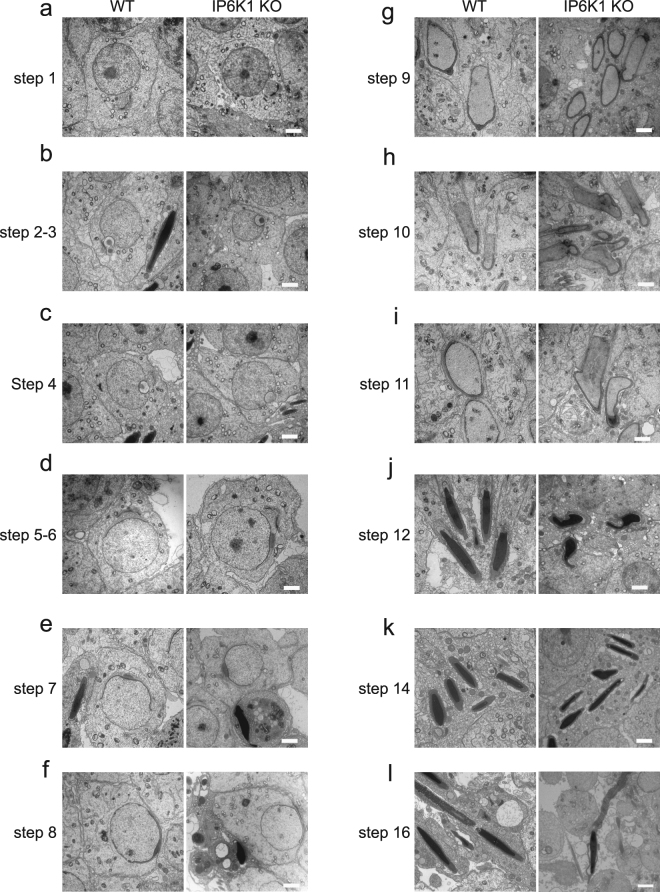
IP6K1 deletion caused disorientation and malformation of elongating/elongated spermatids. (**a–l)** Electron microscopy showed steps 1–16 spermatids from wild type and *IP6K1* KO mice. (**a**,**b**,**c**,**d**,**e**,**f**) Steps 1 to 8 round spermatids of *IP6K1* KOs displayed relatively normal morphology. (**g**,**h**,**i**,**j**,**k**,**l**) Steps 9 to 16 elongating/elongated spermatids in *IP6K1* KOs were disoriented and malformed Scale bar 2 μm.

**Figure 6:**
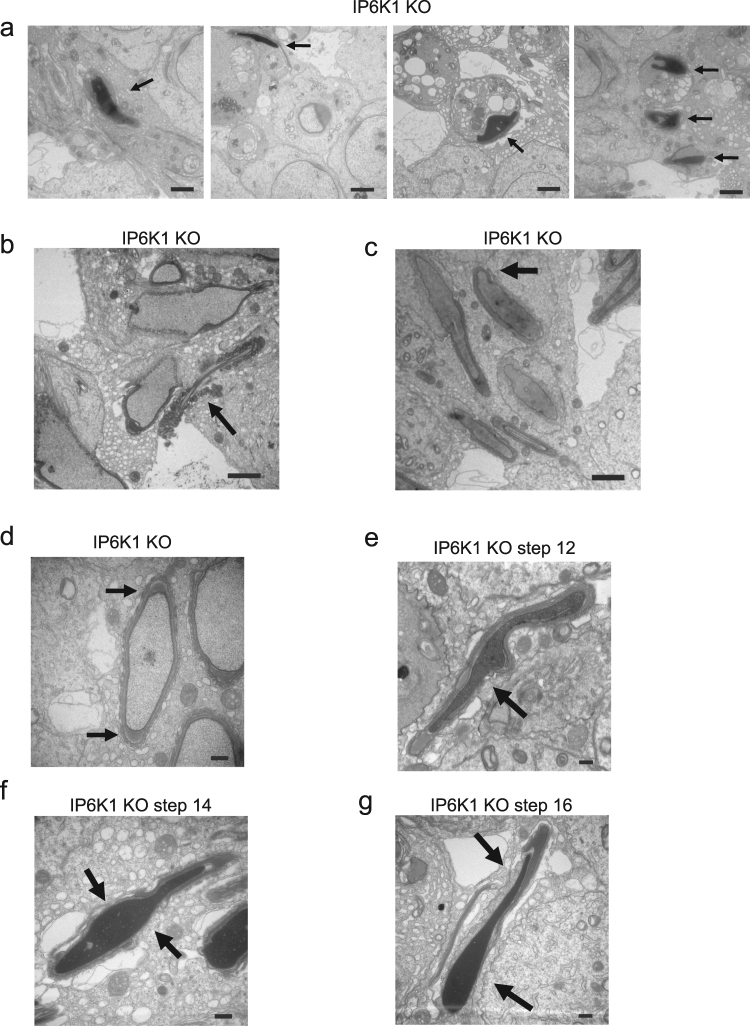
IP6K1 deletion disrupted spermatid cell polarity and caused degeneration of acrosomes. (**a**–**g**) Evaluation of spermatids by electron microscopy. (**a**) Sperm cells were engulfed (arrows) by Sertoli cells in stage VII and stage VIII seminiferous tubules of *IP6K1* KOs. Scale bar 2 μm. (**b**) Severed sperm tails (arrow) showed in stage IX seminiferous tubules. Scale bar 2 μm. (**c**) Disorientation of elongating spermatids (arrow) in *IP6K1* KOs. Scale bar 2 μm. (**d**) Spermatids lost cell polarity in *IP6K1* KOs. Picture shows spermatid with double acrosome granules (arrow). Scale bar 2 μm. (**e**) Disconnected acrosome (arrow) was evident in step 12 spermatids of *IP6K1* KOs. Scale bar 500 nm. (**f**) The integrity of acrosomes was disrupted (arrows) in step 14 spermatids of *IP6K1* KOs. Scale bar 500 nm. (**g**) The step 16 spermatids from *IP6K1* KOs lost a major part of acrosomes (arrows). Scale bar 500 nm.

To further assess mechanisms underlying the abnormalities in *IP6K1* KOs, we evaluated different steps of spermatid differentiation by electron microscopy (Fig. 5). Development of round spermatids was relatively normal in *IP6K1* KOs (Fig. 5a–f). The most notable abnormalities occurred in the disposition of elongating/elongated spermatids at steps 9–16 (Fig. 5g–l). Disorientation and malformation of spermatids was marked at steps 9–16 (Fig. 5g–l), indicating that spermatids lose cell polarity in *IP6K1* KOs. At steps 12–16 in the KOs (Fig. 5j–l), the structure of spermatid heads was abnormal or improperly formed. The step 16 spermatids were not released but eventually engulfed by Sertoli cells in *IP6K1* KO stage VII and VIII seminiferous tubules (Fig. 6a). Severed sperm tails were seen in stage IX of the seminiferous epithelium of *IP6K1* KOs (Fig. 6b).

### IP6K1 deletion disrupts cell polarity and causes malformation of acrosome

We examined cell polarity of the spermatids. In *IP6K1* KO seminiferous tubules, the elongating spermatids lost orientation (Fig. 6c), and some elongating spermatids showed double acrosome granules (Fig. 6d), indicating that cell polarity was disrupted. We examined the structure of elongating/elongated spermatids in *IP6K1* KOs and found malformations of acrosomes (Fig. 6e–g). Disconnected acrosomes were first seen in step 12 spermatids (Fig. 6e). The integrity of the acrosomes gradually degenerated during differentiation in *IP6K1* KOs (Fig. 6f,g).

### IP6K1 deletion disrupts apical ectoplasmic specialization

**Figure 7:**
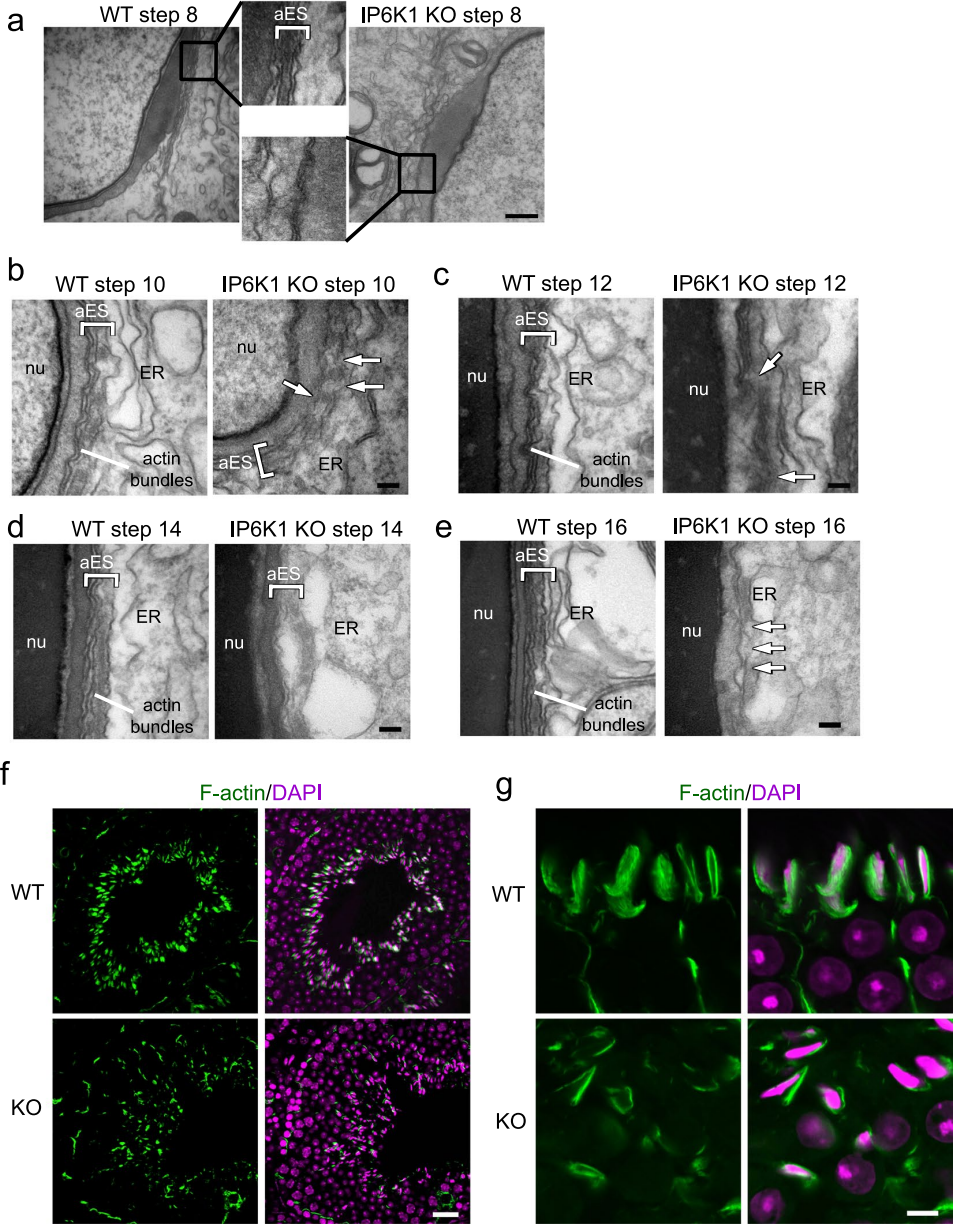
IP6K1 deletion disrupted apical ectoplasmic specialization. (**a–e**) Evaluation of spermatids by electron microscopy. (**a**) No characteristic apical ectoplasmic specialization (aES) was observed in step 8 spermatids of *IP6K1* KOs (inset). Scale bar 500 nm. (**b**) The aES was not well formed in step 10 spermatids of *IP6K1* KOs (arrows). ER: endoplasmic reticulum; nu: nucleus. Scale bar 100 nm. (**c**) Disorganized aES was seen in step 12 spermatids of *IP6K1* KOs (arrows). ER: endoplasmic reticulum; nu: nucleus. Scale bar 100 nm. (**d**) Apical ES was collapsing in step 14 spermatids of *IP6K1* KOs. ER: endoplasmic reticulum; nu: nucleus. Scale bar 100 nm. **(e**) Apical ES collapsed in step 16 spermatids of *IP6K1* KOs (arrows). ER: endoplasmic reticulum; nu: nucleus. Scale bar 100 nm. (**f** and **g**) The actin surrounding spermatids were decreased in *IP6K1* KOs. Staining of F-actin by phalloidin on seminiferous tubules. Scale bar 20μm in (f); scale bar 5μm in (g).

We examined the interaction between elongating/elongated spermatids and Sertoli cells, known as the apical ectoplasmic specialization (ES) (Fig. 7a–e). The actin filament bundles that align perpendicular to the Sertoli cell plasma membrane were a characteristic feature of the apical ES in the WT preparations (Fig. 7a–e). At step 8 of spermatid differentiation, the spermatid nuclei made contact with the cell surface. The apical ES was formed between the spermatids and Sertoli cells of WT mice. In *IP6K1* KOs, however, no characteristic apical ES was observed at step 8 (Fig. 7a). Apical ES was observable in step 10–16 spermatids of the *IP6K1* KOs. However, the structures were disrupted. The apical ES was not well formed in step 10–12 spermatids (Fig. 7b,c), appeared to be collapsing in step 14 spermatids of *IP6K1* KOs (Fig. 7d), and collapsed in step 16 spermatids of the *IP6K1* mutants (Fig. 7e).

The staining of F-actin on testes showed that in WT seminiferous tubules, sperm cells covered with abundant actin filaments. In *IP6K1* KOs, however, the cells were only partially associated with actin bundles (Fig. 7f,g).

### IP6K1 deletion disrupts apical tubulobulbar complexes

**Figure 8:**
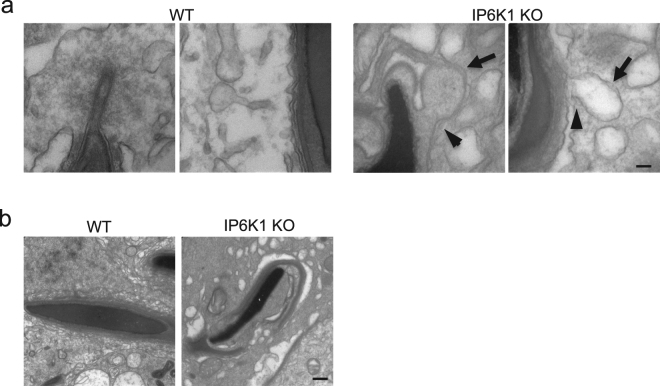
IP6K1 deletion disrupted spermatid-Sertoli cell interactions. (**a**) Electron microscopy showed structures of tubulobulbar complexes of WT and *IP6K1* KOs. The bulbs were enlarged (arrows) and the tubular regions were absent (arrowheads) in the *IP6K1* KOs. Scale bar 100 nm. (**b**) Electron microscopy showed step 16 spermatids from WT and *IP6K1* KO stage VII seminiferous tubules. The KO spermatids showed excess residual cytoplasm and degenerated acrosomes. Scale bar 500 nm.

Sperm release requires normal functioning of apical tubulobulbar complexes^30–32^. We examined the morphology of apical tubulobulbar complexes **(**TBCs) in the *IP6K1* mutants (Fig. 8a). The apical TBCs in the *IP6K1* KOs were malformed with absence of proximal tubules and swelling of the bulbs (Fig. 8a). Thus, the defective apical TBCs lead to spermiation failure (Fig. 4d,e) and excess residual cytoplasm in *IP6K1* KO spermatids (Fig. 8b)^27,32^.

## Discussion

In the present study we have developed insights into the role of IP6K1 in male germ cell development. The infertility of *IP6K1* KO mice is evidently caused by deficits in both quantity and quality of sperm. We observed several defects: 1, sloughing of round germ cells; 2, disorientation and malformation of elongating/elongated spermatids; 3, degeneration of acrosomes; 4, disrupted apical ES and apical TBCs; 5, failure of spermiation in the *IP6K1* KO mice. Eventually sperm were phagocytosed by Sertoli cells, a mechanism which may fully account for the observed sperm deficit^23,24^.

Our results are consistent with previous reports^1,11^, including: the expression of IP6K1 in germ cells; complete loss of fertility in *IP6K1* KO males but normal fertility in females; normal testosterone levels in *IP6K1* KO males; reduced size and weight of testes in *IP6K1* KO mice; absence of mature spermatozoa, and premature release of round cells in *IP6K1* KO epididymides; abnormal morphology and incomplete nuclear condensation in elongating/elongated spermatids.

Germ cells and Sertoli cells interact both physiologically and structurally, processes that are fundamental to male fertility^23,34^. The apical ES is a dynamic structure, which normally organizes and orients the spermatids within the seminiferous epithelium. Fragmentation or malformation of the apical ES is associated with infertility^22,35,36^. The apical ES comprises multiple cytoskeleton proteins, adaptor proteins and signaling proteins^22^. One of the most typical features of the apical ES is the array of actin filament bundles that lie perpendicular to the Sertoli cell plasma membrane and are sandwiched between the cisternae of the endoplasmic reticulum and the Sertoli cell plasma membrane^37,38^. Emerging evidence has shown that junctional dynamics of the apical ES are supported by F-actin and the microtubule based cytoskeleton^25^. In *IP6K1* KOs, the apical ES were not well formed or maintained, the actin bundle of the apical ES was collapsed in later stages of spermatid development. IP6K1′s actions often are mediated by binding with other proteins^5,7,39–41^. We have shown that IP6K1 interacts with α-actinin^5^, which regulates junctions of Sertoli cells and germ cells as well as maintaining the apical ES and spermiation^42^. These interactions with α-actinin may mediate the disrupted apical ES and spermiation in the *IP6K1* mutants. It is also possible that IP6K1 binds with junctional proteins that affect the Sertoli-germ cell interactions.

Apical ES is required for the orientation of spermatids in the seminiferous tubules. The abnormal positioning of spermatid heads of *IP6K1* KOs within the epithelium is likely due to defects in the apical ES^43^. The apical ES also plays a role in shaping the spermatid head^44,45^. The malformation of elongated spermatids in *IP6K1* KOs might be the result of defective apical ES and apical TBCs. Thus, the observed aberrations in *IP6K1* KOs, such as dislodged round spermatids and malformation of elongating/elongated spermatids, might reflect defective interactions between spermatids and Sertoli cells. On the other hand, the acrosomal and nuclear shape changes in *IP6K1* KOs may also derive from deficits of the Golgi complex. Acrosomal formation requires active trafficking from the Golgi apparatus^46^. IP6K1 regulates dynein-dependent trafficking pathways, and cells lacking IP6K1 display defects in Golgi maintenance^47^.

Apical TBCs develop at spermatogenic stage VII, and reflect interactions between maturing spermatids and Sertoli cells. Components of apical TBCs include actin filaments, actin binding proteins, adhesion molecules and endocytic proteins^29^. Apical TBCs remove cytoplasm from spermatids and eliminate adhesion junctions between spermatids and Sertoli cells^26,27,32^. Defective apical TBCs fail to remove spermatid-Sertoli cell adhesions, which leads to spermiation failure^30–32,48^. In *IP6K1* KO mice the apical TBCs were malformed with absence of proximal tubules and swelling bulbs, which may account for spermiation failure.

In *IP6K1* KOs, the interactions between spermatids and Sertoli cells were not well formed and prematurely collapsed. Deletion of IP6K1 elicited malformation of elongating/elongated spermatids. This degeneration would also eventually lead to engulfment of the spermatids by the Sertoli cells, and thereby contribute to spermiation failure^18,19^.

IP6K1 regulates endocytic trafficking^49^. Disruption of endocytosis in *EHD4* KO mice results in male infertility similar to that of *IP6K1* KO mice^50^. Accordingly, the deficits of male germ cell development in *IP6K1* KOs may reflect defects of protein trafficking in membranes of the apical ES and apical TBCs.

5-IP7 affects cell-cell adhesion and adhesion-dependent signaling^3,6^. It is uncertain whether the male infertility in *IP6K1* KOs is dependent on 5-IP7 or IP8, both products of IP6K1. The IP6K inhibitor TNP does not induce infertility in mice, which may reflect its inability to penetrate the blood-testis barrier^51^.

In this study, we isolated motile sperm cells after euthanizing mice with CO_2_. This may not be an optimal way to obtain sperm for motility assessment, as CO_2_ inhibits sperm motility^52^. Nonetheless, the deficits of *IP6K1* KO sperm cells were so striking that they are unlikely to derive primarily from CO_2_ treatment.

Non-obstructive azoospermia is a common form of male infertility. Specific molecular mechanisms underlying the majority of these cases have been elusive. Our present findings, as well as the observations of Bhandari and colleagues^11^ indicate that loss of IP6K1 affects multiple steps of sperm development. Thus IP6K1 may be a marker for male infertility with prognostic value.

## Materials and Methods

### Antibodies

IP6K1 (HPA040825), IP6K2 (HPA007532) and IP6K3 (SAB4500277) antibodies were from Sigma-Aldrich. Alexa Fluor^TM^ 488 Phalloidin (A12379) was from Thermo Fisher Scientific. GATA-4 (sc-25310) and calretinin (sc-365956) antibodies were from Santa Cruz Biotechnology. Acetyl-α-Tubulin antibody (5335) was from Cell Signaling Technology.

### Animals

*IP6K1*, *IP6K2 and IP6K3* KO mice (C57BL/6) were generated by heterozygous breeding^2,10^. The wild-type and *IP6K1* KO mice were littermates. *IP6K2*/*IP6K3* double knockout mice were generated by breeding *IP6K2* KOs with *IP6K3* KOs. Animal breeding and procedures were conducted in strict accordance with the NIH Guide for Care and Use of Laboratory Animals and were approved by the Johns Hopkins University Committee on Animal Care. Mice at 10 weeks old were utilized in experiments. The investigation conformed to the Guide for the Care and Use of Laboratory Animals published by the US National Institutes of Health.

### Mice sexual behavior

Male mouse sexual behavior was evaluated by examining the female’s face and anogenital region. For analysis of fertility after normal mating, male mice were housed with females for 3 months. Vaginal plugs, pregnancies, and pups were recorded. Copulatory ability was determined by the appearance of vaginal plugs.

### Isolation of motile sperm cells and spermatids

Male mice were euthanized by CO_2_. Motile sperm cells were obtained by gently squeezing the excised epididymides; cells were collected in Krebs–Ringer solution. For collecting spermatids from testes, the testes were minced and forcefully mixed; the dislodged spermatids were collected in Krebs-Ringer solution.

### Immunofluorescence staining

Wild-type and *IP6K1* KO mice were euthanized, then perfused and fixed with 4% (wt/vol) paraformaldehyde. The sections were cut 30 μm thick. The slides were blocked with 10% goat serum for 10 min, then incubated with primary antibodies (1:100 dilution) at 4 °C overnight. The fluorescence labeled secondary antibody (1:500 dilution) was incubated for 1 h at room temperature. Nuclei were counterstained with DAPI. Slices were mounted with ProLong Gold Antifade Mountant. Pictures were taken with a confocal microscope (Zeiss LSM 700, NIH Grant# S10 OD016374).

### Hematoxylin and eosin staining

Wild-type and *IP6K1* KO mice were euthanized, then perfused and fixed with 4% (wt/vol) paraformaldehyde. Testes and epididymides were excised and embedded in paraffin. Sections were cut 5 μm thick and stained with hematoxylin and eosin following standard procedures.

### Blood FSH, LH and testosterone

Blood samples from wild type and *IP6K1* KO mice were collected. The plasma concentrations of FSH and LH were determined by using a mouse FSH ELISA kit (Neobiolab) and a mouse LH ELISA kit (Neobiolab). The plasma concentrations of testosterone were determined by using a testosterone ELISA kit (Enzo Life Sciences).

### Transmission electron microscopy and Toluidine blue staining

Wild-type and *IP6K1* KO mice were euthanized, then perfused and fixed with buffer containing 2% (wt/vol) glutaraldehyde, 2% (wt/vol) paraformaldehyde, 100 mM sodium cacodylate and 3 mM MgCl_2_, pH 7.4. Testes were excised and post-fixed in 1.5% potassium ferrocyanide reduced 2% osmium tetroxide in 100 mM sodium cacodylate with 3 mM MgCl_2_ for 2 h on ice in the dark. After a brief rinse in 100 mM maleate buffer containing 3% sucrose, samples were placed in 2% uranyl acetate in maleate/sucrose for 1 h at 4 °C with slow rocking in the dark. Samples were then dehydrated through a graded series of ethanol, transferred through propylene oxide and were embedded in Eponate 12 Resin and cured at 60 °C for two days.

For transmission electron microscopy, sections were cut 80 nm thick and stained with uranyl acetate followed by lead citrate. Grids were viewed on a Hitachi 7600 TEM operating at 80 kV and digital images captured with an XR50 (5 megapixel) CCD camera.

For Toluidine blue staining, sections were cut 0.5 μm thick and stained with 1% toluidine blue and 1% sodium borate for 1 min.

### Statistical analysis

Quantitative data are expressed as means ± SEM. Data were analyzed by unpaired Student’s t-test. P < 0.05 was considered statistically significant. For non-quantitative data, results were representative of at least 3 independent experiments.

### Data Availability

All data generated or analysed during this study are included in this published article (and its Supplementary Information files).

## Electronic supplementary material

supplementary information
