# Supplementary material for: Multiple aspects of male germ cell development and interactions with Sertoli cells require inositol hexakisphosphate kinase-1
Source: Sci Rep. 2018 May 4;8:7039. doi: 10.1038/s41598-018-25468-8 (PMC5935691; doi:10.1038/s41598-018-25468-8)
Supplement: Supplementary file 1 — supplementary information [file 41598_2018_25468_MOESM1_ESM.pdf]

Title: Multiple aspects of male germ cell development and interactions with Sertoli cells require inositol hexakisphosphate kinase-1

Authors: Chenglai Fu[1,2]*, Tomas Rojas[2], Alfred C. Chin[2], Weiwei Cheng[3], Isaac A. Bernstein[2], Lauren K. Albacarys[2], William W. Wright [4] and Solomon H. Snyder[2,5,6]*

Affiliations:

[1]Department of Physiology and Pathophysiology, Tianjin Medical University, Tianjin 300070, China.

[2]The Solomon H. Snyder Department of Neuroscience, Johns Hopkins University School of Medicine, Baltimore, MD 21205, USA

[3]Division of Neuropathology, Department of Pathology, Johns Hopkins University School of Medicine, Baltimore, MD 21205, USA

[4]Department of Biochemistry and Molecular Biology, Johns Hopkins Bloomberg School of Public Health, Baltimore, MD 21205, USA

[5]Department of Pharmacology and Molecular Sciences, Johns Hopkins University School of Medicine, Baltimore, MD 21205, USA

[6]Department of Psychiatry and Behavioral Sciences, Johns Hopkins University School of Medicine, Baltimore, MD 21205, USA

*Corresponding author. Email: cfu9@jhmi.edu or ssnyder@jhmi.edu

S1

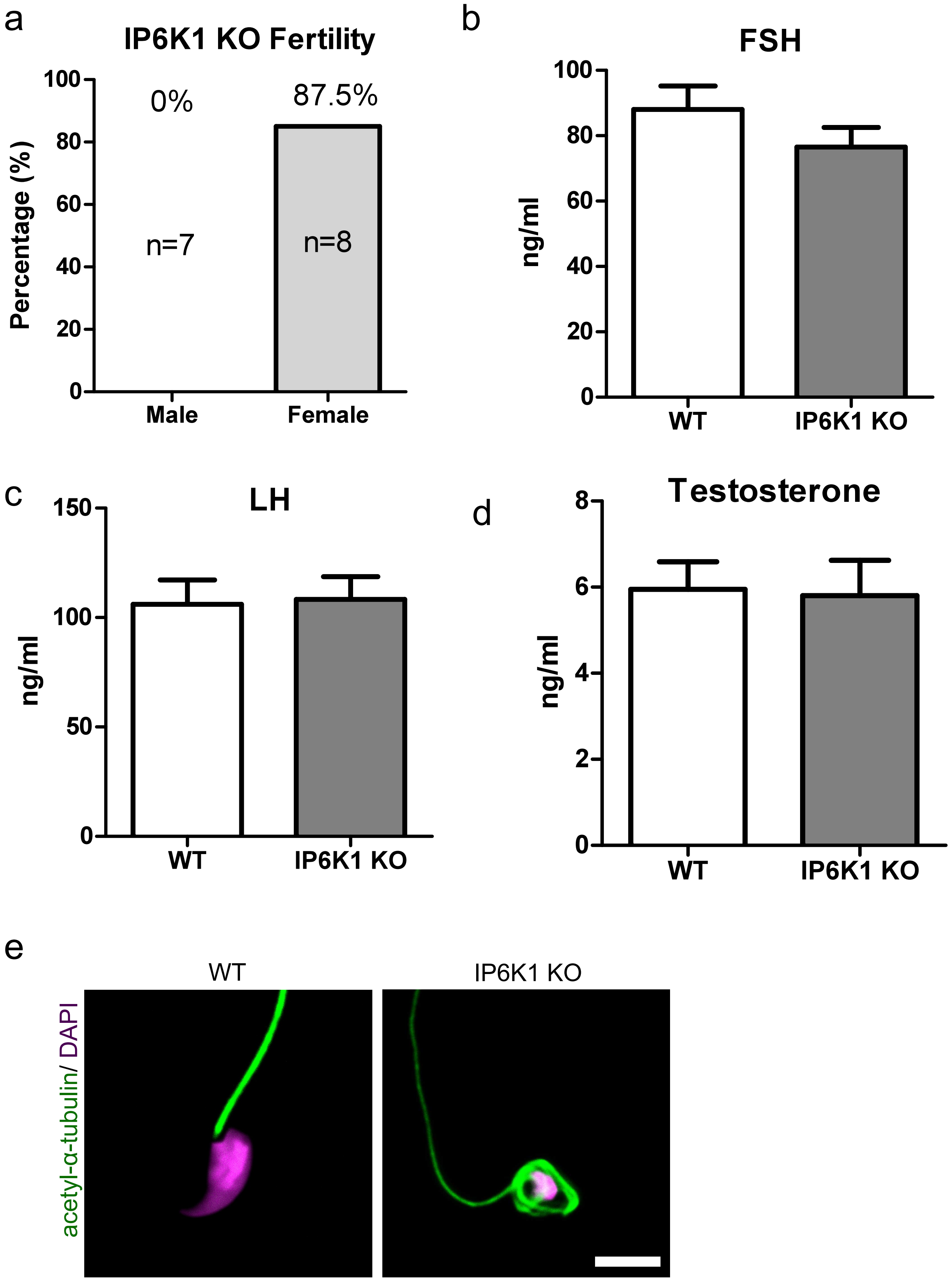
a
IP6K1 KO Fertility
0%
87.5%
n=7
n=8
Percentage (%)
100
80
60
40
20
0
Male
Female
b
FSH
ng/ml
100
80
60
40
20
0
WT
IP6K1 KO
c
LH
ng/ml
150
100
50
0
WT
IP6K1 KO
d
Testosterone
ng/ml
8
6
4
2
0
WT
IP6K1 KO
e
WT
IP6K1 KO
acetyl-α-tubulin/ DAPI

Fig. S1 *IP6K1* KO male mice were infertile. (*a*) *IP6K1* KO male mice (n=7) mated with wild type female mice, none of the females were pregnant; *IP6K1* KO female mice (n=8) mated with wild type male mice, Seven of 8 *IP6K1* KO females were pregnant and produced progeny. (*b*) Normal blood FSH levels in *IP6K1* KO male mice, determined by ELISA. (n=6, male 10 weeks old, unpaired student's t-test, mean ± s.e.m.). (*c*) Normal blood LH levels in *IP6K1* KO male mice, determined by ELISA. (n=6, male 10 weeks old, unpaired student's t-test, mean ± s.e.m.). (*d*) Normal blood testosterone levels in *IP6K1* KO male mice, determined by ELISA. (n=6, male 10 weeks old, unpaired student's t-test, mean ± s.e.m.). (*e*) Sperm cells were isolated from epididymides. Immunostaining of acetyl-α-tubulin for sperm tails; and nucleus was stained by DAPI. Abnormal sperm heads coiled by sperm tails were seen in the *IP6K1* KOs. Scale bar 5 μm.

S2

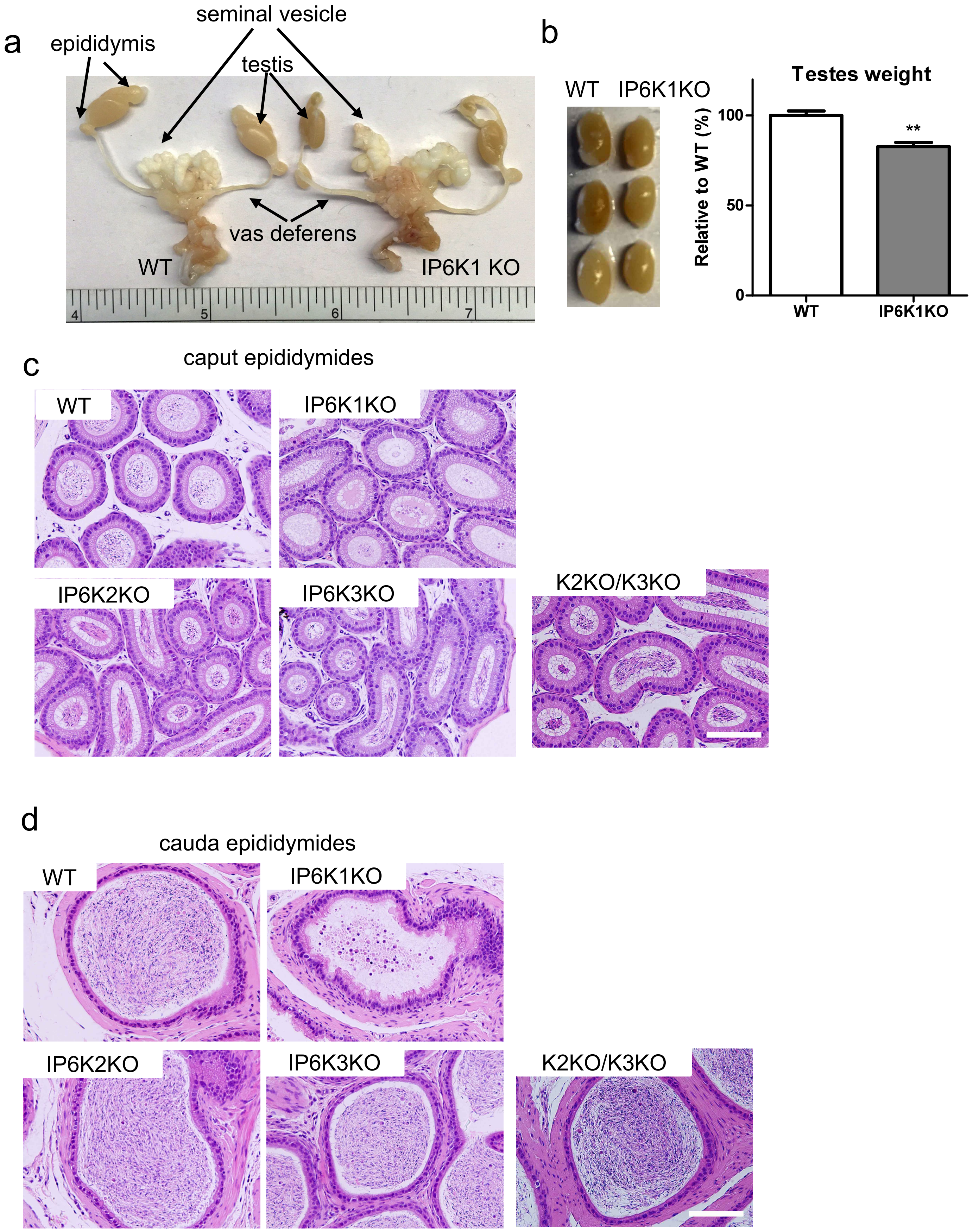
a
epididymis
seminal vesicle
testis
vas deferens
WT
IP6K1 KO
4
5
6
7
b
WT
IP6K1KO
Testes weight
Relative to WT (%)
100
50
0
**
WT
IP6K1KO
c
caput epididymides
WT
IP6K1KO
IP6K2KO
IP6K3KO
K2KO/K3KO
d
cauda epididymides
WT
IP6K1KO
IP6K2KO
IP6K3KO
K2KO/K3KO

Fig. S2 *IP6K1* KO testes were smaller and devoid of sperm in epididymides.(*a*) Image shows the reproductive organs of *IP6K1* WT and KO male mice; The *IP6K1* KO male mice have intact reproductive organs but smaller testes. (*b*) Testes of *IP6K1* KO mice were smaller than those of wild type (n=6). **P<0.01 by unpaired student's t-test. (*c*) H&E staining of caput epididymides of wild type, *IP6K1* KO, *IP6K2* KO, *IP6K3* KO and *IP6K2/IP6K3* double knockout mice. *IP6K1* KO caput epididymides were devoid of sperms. Scale bar 100 μm. (*d*) H&E staining of cauda epididymides of wild type, *IP6K1* KO, *IP6K2* KO, *IP6K3* KO and *IP6K2/IP6K3* double knockout mice. Round germ cells were seen in *IP6K1* KO cauda epididymides. Scale bar 100 μm.

S3

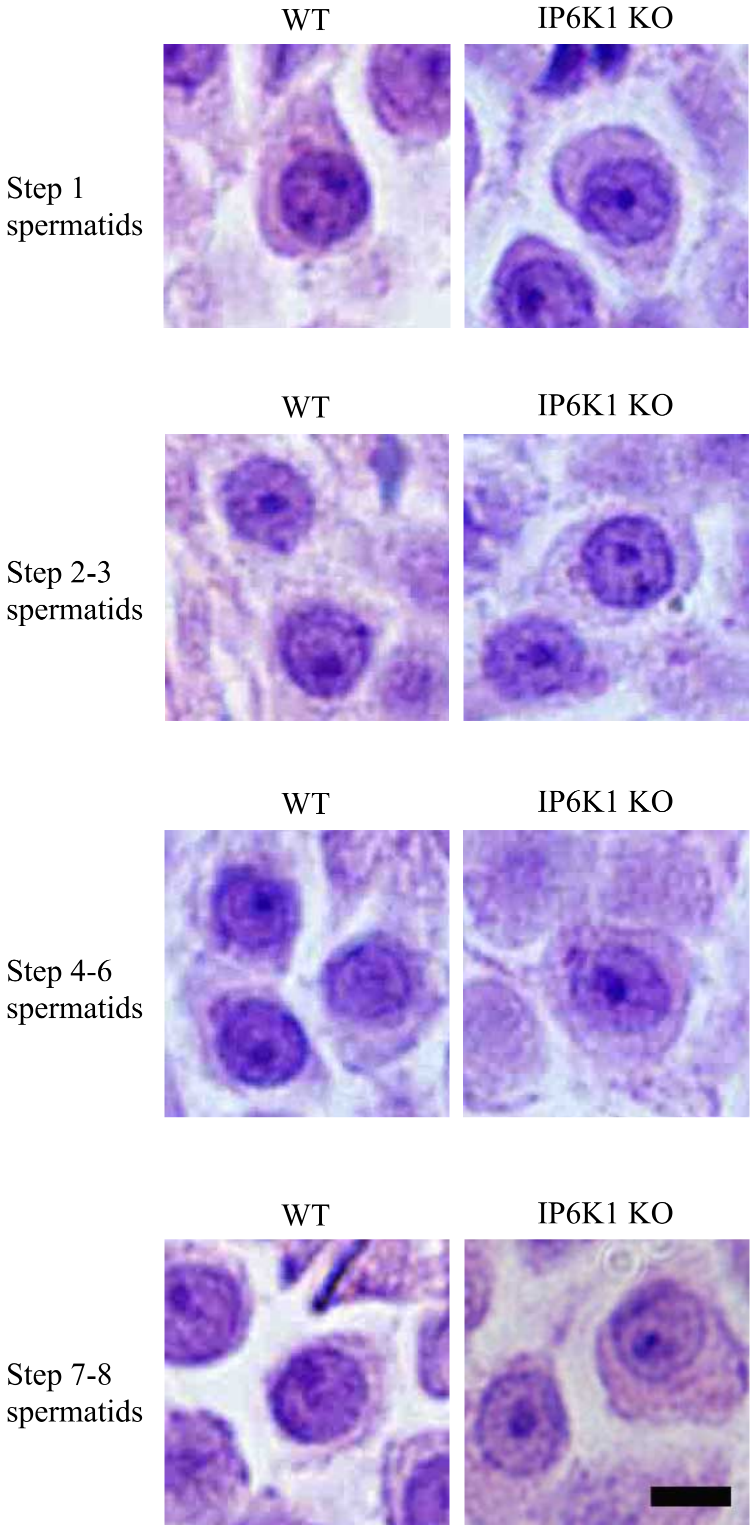
WT
IP6K1 KO
Step 1
spermatids
WT
IP6K1 KO
Step 2-3
spermatids
WT
IP6K1 KO
Step 4-6
spermatids
WT
IP6K1 KO
Step 7-8
spermatids

Fig. S3 H&E staining of WT and *IP6K1* KO testes revealed morphologically normal round spermatids (steps 1-8) in *IP6K1* KOs. Scale bar 5 μm.

S4

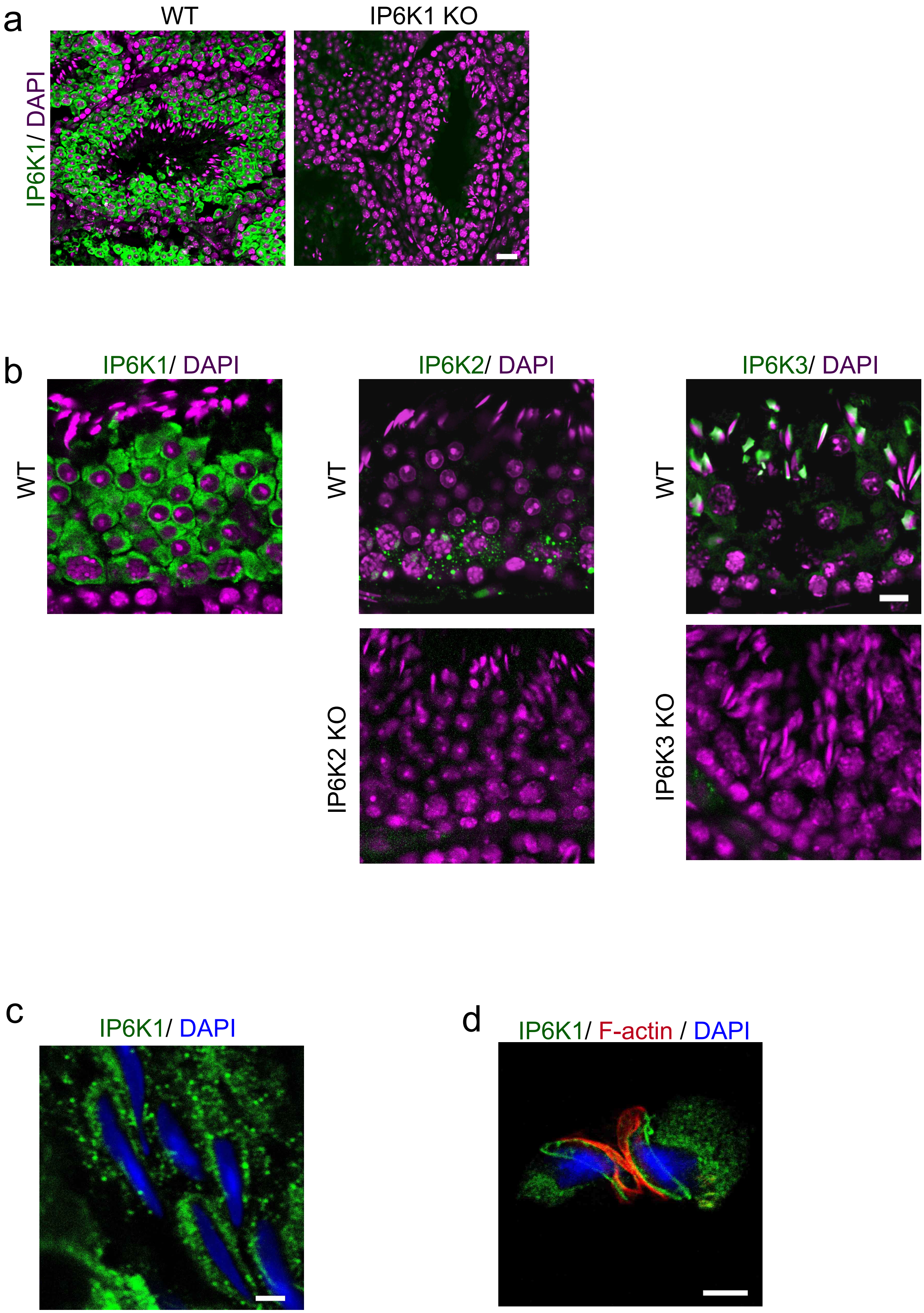
a
WT
IP6K1 KO
IP6K1/ DAPI
b
IP6K1/ DAPI
IP6K2/ DAPI
IP6K3/ DAPI
WT
WT
WT
IP6K2 KO
IP6K3 KO
c
IP6K1/ DAPI
d
IP6K1/ F-actin / DAPI

Fig. S4 IP6K1 was expressed in germ cells. (*a*) Immunostaining of IP6K1 in wild type and *IP6K1* KO testes, scale bar 20 μm. (*b*) Immunostaining of IP6K1 in wild type testes; Immunostaining of IP6K2 in wild type and *IP6K2* KO testes; Immunostaining of IP6K3 in wild type and *IP6K3* KO testes; scale bar 10 μm. (*c*) Immunostaining of IP6K1 in wild type testes, scale bar 2 μm. (*d*) Immunostaining of IP6K1 on loose spermatids released from the minced testes, scale bar 5 μm.

S5

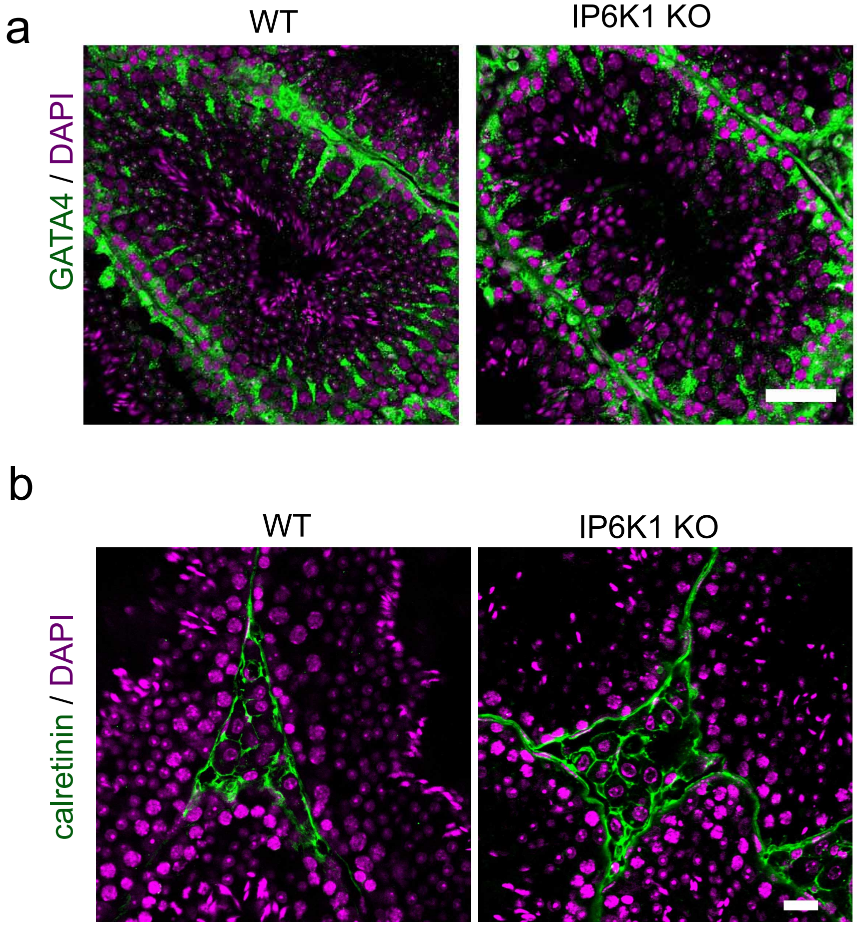

Fig. S5 Relative normal numbers of Sertoli cells and Leydig cells in *IP6K1* KOs. (*a*) Immunostaining of GATA4 for Sertoli cells in WT and *IP6K1* KO testes. DAPI stains nucleus. Scale bar 50 µm. (*b*) Immunostaining of calretinin for Leydig cells in WT and *IP6K1* KO testes. DAPI stains nucleus. Scale bar 20 µm.
